# Editorial: Lipids, lipoproteins and COVID-19

**DOI:** 10.3389/fcvm.2023.1293249

**Published:** 2023-11-01

**Authors:** Mualla Ozcan, Xuewei Zhu, Hanrui Zhang, Ali Javaheri

**Affiliations:** ^1^ Cardiovascular Division, Department of Medicine, Washington University School of Medicine, St. Louis, MO, United States; ^2^ Department of Medicine, Wake Forest University School of Medicine, Winston-Salem, NC, United States; ^3^ Cardiometabolic Genomics Program, Division of Cardiology, Department of Medicine, Columbia University Irving Medical Center, New York, NY, United States; ^4^ Department of Medicine, John J. Cochran Veterans Affairs Medical Center, St. Louis, MO, United States

**Keywords:** lipids, cardiovascular, COVID-19, SARS-CoV-2, lipoprotein, apolipoprotein, cerebrovascular

**Editorial on the Research Topic** Lipids, Lipoproteins and COVID-19

## Introduction

Coronavirus disease-19 (COVID-19), caused by Severe Acute Respiratory Syndrome Coronavirus 2 (SARS-CoV-2), not only impacts the respiratory system but often exhibits extrapulmonary involvement, resulting in systemic disease (1). Existing studies suggest a complex interplay between the virus and various organ systems, including cardiovascular, cerebrovascular, gastrointestinal, musculoskeletal, endocrine, and renal systems.

While the mechanisms driving these pulmonary and extrapulmonary manifestations of COVID-19 remain under investigation, dysregulated immune responses and coagulation abnormalities characterized by hypercoagulation and microthrombosis, may play significant roles. Additionally, numerous studies have pointed to a potential connection between lipid levels and disease severity, suggesting a prognostic and/or therapeutic utility of lipids and lipoproteins in COVID-19.

## Cardiovascular disease and COVID-19

COVID-19 and cardiovascular disease have a bidirectional relationship. On one hand, preexisting cardiovascular risk factors such as hypertension and diabetes, along with chronic cardiovascular conditions, predispose to severe disease. Conversely, COVID-19 can lead to cardiovascular complications, including acute heart failure, arrhythmias, venous thromboembolism, cardiogenic shock, arterial thrombosis, myocardial ischemia or infarction, and myocarditis (2, 3). Moreover, COVID-19 can increase biomarkers of myocardial injury (3–5), but often in the absence of overt cardiac symptoms. The cardiovascular system involvement in COVID-19 has important consequences during recovery from infection and the development of long COVID (1).

Despite the evidence of acute cardiac manifestations of COVID-19, a study by Matejova et al. revealed that in patients with a history of hospitalization due to COVID-19, one-year follow-up echocardiography indicated only a subtle left ventricle diastolic impairment and left atrial enlargement, which was not clinically significant. On the other hand, Bürgi et al. observed a significant increase in high-sensitivity troponin I levels among men aged 54 or older after infection, and these elevated levels persisted for at least 14 months, suggesting a potential ongoing myocardial injury, albeit without clear clinical significance. These findings shed valuable light on the need for future long-term studies to investigate cardiovascular outcomes of COVID-19.

## Cerebrovascular disease and COVID-19

Stroke and other cerebrovascular events appear to be uncommon complications of COVID-19, but when they occur, they can significantly increase morbidity and mortality. Moreover, a study by De Michele et al. demonstrated that COVID-19 extends the infarct volume during acute ischemic stroke (AIS), one of the feared complications of COVID-19. Though the mechanisms underlying AIS are not yet fully known, it is thought to be driven by multiple pathophysiological factors, including hypercoagulation and microthrombosis. De Michele et al. demonstrated that COVID-19 increases biomarkers of endothelial dysfunction and hence plays a major role in endothelial activation, which can potentially explain the increased risk and severity of AIS in COVID-19.

## Lipids and COVID-19

Studies have consistently observed alterations in lipid profiles in patients with COVID-19. Total cholesterol, low-density lipoprotein cholesterol (LDL-C), high-density lipoprotein cholesterol (HDL-C), cholesterol metabolite 27-hydroxycholesterol, and apolipoprotein M, B and A-I levels often decrease in patients with COVID-19, similar to trends seen in other infections (6–8). Chidambaram et al. demonstrated that the decreases in total cholesterol and HDL-C, measured at the time of admission, were more pronounced in patients with severe disease and those who did not survive. Hence, they suggested that lipids may serve as biomarkers for predicting disease severity and mortality. In addition to HDL-C, Mietus-Snyder et al. highlighted the significance of other lipid particles, including total, large, and small HDL particles, as well as HDL functional cholesterol efflux capacity (CEC), in relation to the severity of COVID-19 among pediatric patients. Consequently, they have suggested a potential prognostic role of HDL parameters in COVID-19, particularly among youth.

Mechanistically, cholesterol in the host cell plasma membrane influences the entry of SARS-CoV-2. Serum lipids, particularly LDL-C and HDL-C, are in constant interaction with the lipid rafts in the host cell membranes and can modulate virus-host cell interactions and disease severity (Chidambaram et al., 9, 10). Adipose tissue has also been implicated in contributing to the hyper-inflammatory state seen in severe COVID-19 cases and being responsible for their poor prognoses, including death (11, 12).

Apolipoproteins have emerged as predictive biomarkers for various diseases, including COVID-19 (8, 13). Supporting this, Mietus-Snyder et al. demonstrated that a decrease in apolipoprotein A-I is associated with increased clinical severity in COVID-19. Additionally, both preclinical and clinical studies indicated a potential therapeutic role of apolipoproteins and agents targeting them in various disease settings, including COVID-19 (13–16). Apolipoproteins influence human vascular biology and atherosclerotic cardiovascular disease (17). Hence, one of the potential mechanistic hallmarks underlying the benefits of apolipoproteins is suggested to be protection against COVID-19-induced endothelial dysfunction.

## Lipid-lowering therapy and COVID-19

In patients with COVID-19, the most severe complication is sepsis. While LDL-C is a well-known risk factor for coronary heart disease, it is noteworthy that both LDL-C and HDL-C play protective roles against infection and sepsis. A study conducted by Felici et al. revealed significant reductions in LDL-C and HDL-C in patients with sepsis and these derangements persisted in long-term after recovery from sepsis. Interestingly, Gong et al. showed that both low and high LDL-C levels are associated with an increased risk of severe COVID-19. Consequently, lipid-lowering therapy should be performed cautiously as plasma LDL-C levels could potentially have a dual impact on these patients, similar to a double-edged sword.

Statins, known as 3-hydroxy-3-methylglutaryl coenzyme A reductase (HMG-CoA) inhibitors, are well-known lipid-lowering drugs, decreasing LDL-C levels. Beyond their cholesterol-lowering effects, they have also been suggested to have a wide range of pleiotropic effects, including anti-inflammatory, antithrombotic, and antioxidant effects. Kouhpeikar et al. demonstrated that statins decreased the composite outcomes of mortality, ICU admissions, and intubations among COVID-19 patients. Statin treatment also lowered inflammatory markers, including C-reactive protein (CRP) levels and neutrophil counts. These findings suggest a potential anti-inflammatory role of statins in mitigating the composite adverse outcomes associated with COVID-19.

## Conclusion

In conclusion, the articles published in this research topic hold immense importance in understanding the pathophysiology, diagnosis, prognosis, and treatment of COVID-19 (Matejova et al., Bürgi et al., De Michele et al., Chidambaram et al., Mietus-Snyder et al., Felici et al., Gong et al., Kouhpeikar et al.). Further research is necessary to establish a theoretical and clinical foundation for using lipids as biomarkers for both COVID-19 and sepsis prognosis. Additionally, the potential role of lipid-lowering therapies and apolipoproteins in COVID-19 treatment warrants further thorough investigation.
